# The Role of the Results of Functional Tests and Psychological Factors on Prediction of Injuries in Adolescent Female Football Players

**DOI:** 10.3390/ijerph19010143

**Published:** 2021-12-23

**Authors:** Ulrika Tranaeus, Andreas Ivarsson, Urban Johnson, Nathan Weiss, Martin Samuelsson, Eva Skillgate

**Affiliations:** 1 Department of PNB, The Swedish School of Sport and Health Sciences, 144 86 Stockholm, Sweden; 2 Unit of Intervention and Implementation Research for Worker Health, Institute of Environmental Medicine, Karolinska Institutet, 171 77 Stockholm, Sweden; nathan.weiss@shh.se (N.W.); eva.skillgate@shh.se (E.S.); 3 Center of Research on Welfare Health and Sport, Halmstad University, 301 18 Halmstad, Sweden; andreas.ivarsson@hh.se (A.I.); urban.johnson@hh.se (U.J.); 4 Department of Sport Science and Physical Education, University of Agder, 4630 Kristiansand, Norway; 5 Department of Health Promotion Science, Musculoskeletal & Sports Injury Epidemiology Center, Sophiahemmet University, 114 86 Stockholm, Sweden; 6 Naprapathögskolan—Scandinavian College of Naprapathic Manual Medicine, 114 19 Stockholm, Sweden; samuelssonmartin@live.se

**Keywords:** athletic injury, coping, girls, soccer

## Abstract

Football is a popular sport among adolescent females. Given the rate of injuries in female footballers, identifying factors that can predict injuries are important. These injuries are often caused by complex reasons. The aim of this study was to investigate if the combination of demographic (age, number of training and match play hours/week), psychosocial (perceived stress, adaptive coping strategies) and physiological factors (functional performance) can predict a traumatic injury in adolescent female footballers. A cohort consisting of 419 female football players aged 13–16 years was established. Baseline questionnaires covered potential risk factors for sport injuries, and measurements included football-related functional performance tests. Data were collected prospectively with a weekly online questionnaire for 52 weeks covering, e.g., injuries, training, and match play hours/week. A total of 62% of the players reported at least one traumatic injury during the 52 weeks. The coping strategy “positive reframing” had the strongest association with the risk of traumatic injuries. The combination of more frequent use of the coping strategy, positive reframing, and high levels of physical performance capacity may prevent a traumatic injury in adolescent female footballers. Coaches are encouraged to adopt both physiological and psychological factors when preventing injuries in young female footballers.

## 1. Introduction

Football is a popular sport for females, and adolescent females under 17 years old represent 62.5% of all female players according to the International Football Federation (FIFA). Injuries are the back coin of sports, and female footballers are no exception. The incidence of injuries in female players was 6.30 (95% CI 5.40–7.36) per 1000 player hours, divided into traumatic injuries 3.19 (95% CI 2.57–3.97) and overuse injuries 2.84 (95% CI 2.25–3.57) during five seasons [1]. The injury incidence during matches is six times higher than the incidence during training: 19.2 injuries per 1000 h of exposure to matches (95% CI 16.0–22.4) and 3.5 injuries per 1000 h of training (95% CI 2.4–4.6) in adult female football players [2]. The most common injury localizations in females are the knee, ankle, and thigh [1,3,4,5,6,7]. Risk factors and injury mechanisms for these injuries have been commonly investigated. Most studies investigate and report isolated risk factors such as previous injury [8], a hamstring/quadriceps ratio less than 55%, and results of plyometric tests, e.g., poor performance in a drop jump landing test, which is associated with increased risk of ankle injury [9]. Other identified risk factors are young age [7,10], physical complaints in the beginning of the season [10], and lower level of preseason aerobic fitness [11]. Another type of physiological factors that have been discussed in relation to injury risk is functional test performance. More specifically, results from functional tests performed in preseason combined with internal and external characteristics in young male football players showed no association to risk for injury [12]. However, the results of functional performance tests in young male and female team players showed significant differences between injured and uninjured players, independent of sex [13]. Results from functional screening tests have been suggested to predict injuries in both male and female footballers and from adolescent players to senior elite players [14].

It is most likely that injuries have a complex etiology and mechanisms [15]. More specifically, it is suggested that psychophysiological stressors, in combination with physiological mechanisms and behavioral mechanisms, influence health outcomes, i.e., increased injury and illness incidence, exercise training adaption and prolonged injury rehabilitation [15]. To explain the potential relationship between psychosocial factors and injury risk, several theoretical frameworks have been developed. An often-cited framework is “The stress and injury model” [16]. Within this model it is suggested that psychosocial risk factors influence an athlete’s stress response [16]. The magnitude of the stress response depends on the athlete’s personality, history of stressors and coping resources and the interaction between these factors. History of stressors and stress responses are identified to have the strongest association with injury rates [17]. In football, daily hassles [18], stress from teammates and coaches [19,20,21], and ineffective coping [18] are all identified as potential risk factors. Hence, coping strategies are suggested to influence the perceived stress which, in turn, influence the susceptibility for injuries [22].

From a biopsychosocial perspective, the research area identifying risk factors is suggested to change from determining single risk factors for injuries to identifying several factors which may interact and build a complex system that contribute to risk for injuries [23]. Such a complex system of related risk factors for injuries may also include demographic variables such as age, sport, training hours, etc. Complex systems can be built of factors from different domains that might interact and potentially lead to injuries (e.g., neuromuscular control [24,25], experience of stress, and coping strategies) [15,16].

The use of interrelated factors that can explain injury mechanisms also increases the possibility of developing preventive strategies [23]. Many studies have been aiming to prevent sport injuries [26,27], however, in women’s football, there is hitherto low-level evidence that injury prevention programs reduce injuries [28].

To overcome limitations in previous research, we adapted a biopsychosocial perspective when selecting potential risk factors for the study. A biopsychosocial approach includes complex and multifactorial risk factors which need to be analyzed and evaluated using different (combinations of) interrelations. However, information about all possible factors cannot be collected and analyzed in one study, and the need for longitudinal studies with several potential risk factors are suggested to identify risk patterns [23]. Additionally, a second limitation is that most previous studies have not applied statistical analyses where nonlinear interactions between risk factors can be included (e.g., Ivarsson and Stenling, 2019 [29]).

The aim of this study was to investigate if the combination of demographic (i.e., age, number of training and match play hours/week), psychosocial (i.e., perceived stress, adaptive coping strategies) and physiological factors (i.e., functional performance) can predict a traumatic injury in adolescent female football players.

## 2. Materials and Methods

This study is part of a prospective observational cohort study designed in agreement with Strengthening the Reporting of Observational Studies in Epidemiology (STROBE) guidelines [30].

### 2.1. Participants

A cohort consisting of 419 adolescent female football academy players was established. The adolescent female football players were 12–17 years old (mean = 13.9, SD 1.1) and had played football for an average of seven years (SD 2.2) see Table 1. Clubs were contacted and invited to the study, they were given oral and written information. Clubs with teams who volunteered to take part in the study were provided with detailed oral and written information in the presence of players, legal guardians, and coaches.

### 2.2. Procedure

A baseline questionnaire was answered ahead of appointment for the physical test. One of the test leaders checked that the questionnaires were completed and included written consent from legal guardians. Next, the players participated in physical tests. They trained and competed as usual prior to testing. Players refrained from tests that evoked pain, provoked ongoing injuries or other health-related issues. Prior to performing the physical tests, players completed a standardized seven-minute warm-up program comprising four minutes of jogging, 10 × 1 body weight squats, 10 × 1 body weight squat jumps, and 10 × 1 unilateral body weight lunges. When baseline and physical tests were completed, the follow-up measurements were collected prospectively during one year from the baseline with a weekly online questionnaire sent by email and a reminding text message to the players. In cases when players did not answer the weekly follow-up questions, test leaders visited a training, and the questions were answered with paper and pen at site.

### 2.3. Baseline Measurements

#### 2.3.1. Questionnaires

A baseline questionnaire was provided to the participants ahead of the physical tests covering potential risk factors for the etiology of sport injuries, as well as information about the players’ general health status. Players were surveyed in various areas including (a) health, (b) lifestyle, (c) socioeconomic factors, (d) football-related factors, (e) psychosocial factors, (f) previous injury history, and (g) back and neck pain [31].

The experienced stress was measured with a single–item question using a five-point Likert scale from “Never” to “Most days in a week” (from 1 to 5) [32]. Coping strategies were assessed by a 28 item self-report questionnaire that measure adaptive and maladaptive strategies to coping with stressful events using a four-point Likert scale (Brief COPE) [33]. The Likert scale ranged from 0 “I have not been doing this at all” to 3 “I’ve been doing this a lot”. The Brief COPE covers 14 dimensions, each consisting of two items. In the current study, we decided, however, to only include the eight adaptive coping strategies (i.e., active coping, planning, positive reframing, acceptance, humor, religion, use of emotional support, and use of instrumental support). The included dimensions showed Cronbachs α from 0.57 to 0.82 [33].

#### 2.3.2. Functional Performance Tests

To assess the player’s unilateral jump performance, the One-leg Long Box Jump Test (OLLBJ) and square hop test were performed [34,35]. A 40 × 40 cm square was marked on the floor and used as a reference mark in both tests.

In the OLLBJ, the starting position was calculated by dividing the player’s height (cm) by 1.6 (height/1.6). Thereafter, the player was instructed to stand on one leg on the starting position and then jump on one leg directed inside the boundaries of the square and maintain balance after landing. The players were facing the same way during the test. Three warm-up trials and five consecutive test trials were performed on each leg without rest. The total number of approved trials were registered by the test leader. In this analysis we used the mean results of approved jumps from both legs divided by two.

During the square hop test, the player was instructed to jump on one leg in and out of the square as many times as possible for 15 s in a clockwise direction. This was timed with a stopwatch whilst the test leader registered the number of approved jumps. The player performed two warm-up trials on each foot prior to executing the test.

### 2.4. Follow-Up Measurement and Outcome

Follow-up measurements were collected weekly, prospectively, during one year from the baseline. In the weekly online questionnaire, the players were asked to answer several questions regarding, e.g., new, and ongoing injuries. To assess whether players sustained football related injuries throughout the follow-up period, a modified version of the Swedish OSTRC-O was employed and included in the weekly online questionnaire [36,37]. In this modified version of the OSTRC-O, a question regarding absence/reduced participation in training/match due to reasons not related to injuries were added, and to specify injuries in different anatomical localizations.

Football related injuries reported in OSTRC-O in the weekly online questionnaire leading to moderate or severe reductions in participation/and or sports performance or complete inability to participate in sport were classified as substantial injuries [36]. Players reporting new substantial injuries were contacted by telephone by a clinically experienced research assistant to answer a standardized interview with questions concerning the injury, such as: injury mechanism, localization, type, time-loss, reinjury, diagnosis, and medical care. Injuries are divided into traumatic and gradual onset. The traumatic injury is defined as a result from a specific, identifiable event, whereas injuries with gradual onset are defined as an injury due to repeated microtrauma without a single, identifiable event responsible for the injury [31].

### 2.5. Statistical Analyses

Descriptive analyses were conducted in SPSS (i.e., correlation analyses). Classification and Regression Trees (CRT) were used to test the potential relationships between predictors (in the present study; perceived stress, square hop test, One-leg Long Box Jump Test, active coping, instrumental support, planning, acceptance, emotional support, positive reframing, humor, religion, number of training hours/week, number of match hours/week, age) and the outcome variable (in the present study; traumatic injuries). The aim of the analysis is to search for the predictors that differ the most on the outcome variable [38]. More specifically, this modelling technique that “allow non-linear interactions among predictors, as well as depict and make use of these interactions, have been successful in identifying the subset of risk and predictive factors to explain different outcomes” [23]. In line with the proposals within the complex system approach we used statistical methods where specification of nonlinear relationships between independent variables were possible [23,29].

In the analysis procedure, a decision tree is generated based on an automatic stepwise variable selection, aimed to identify exclusive subgroups within the population. Within the analysis, the data are classified into subgroups based on the variable that best explains the dependent variable. Each subgroup continues to generate more subgroups based on the strongest predictor until the last stopping rule triggers. In CRT, “splitting stops when the relative reduction in error resulting from the best split falls below a pre-specified threshold known as the complexity parameter. Typical values of this parameter are in the range of 0.001–0.05” [39]. We followed the criteria suggested by Machuca et al., (2017) [38] for the analysis, which was performed in SPSS Statistics version 26 (SPSS Inc., Chicago, IL, USA). More specifically, the criteria were that (a) the minimum number of cases in the parent node = 70, and (b) the minimum of cases in the terminal nodes = 35. We applied tree pruning to avoid overfitting, with a maximum acceptable difference in risk between the pruned and the subtree of one standard error. To validate the tree, we applied the tenfold cross-validation application. We treated missing data by surrogated splits. We calculated risk differences (with corresponding 95% confidence intervals) to illustrate the magnitude of difference in proportion of injured players between the subgroups.

### 2.6. Ethics

The Regional Ethical Review Board at Karolinska Institutet, Stockholm, Sweden (2016/1251-31/4), approved the study. All participating players and their parents or legal guardians received written and oral information regarding the study and gave their written informed consent when entering the study. Players under the age of 15 were required to obtain written informed consent from their legal guardians.

We followed the ethical principles of the Helsinki declaration.

## 3. Results

### 3.1. Descriptive Results

The response rate during the 52 weeks was 74% of all reports. Of the participants, 62% registered at least one traumatic injury during the study period. Mean values and standard deviation for all variables are presented in Table 2. Additionally, bivariate correlations between the included independent variables can be found in Table 2. Intraclass correlation for OLLBJ: ICC 0.47–0.90; Square hop: intraclass correlation, ICC 0.40–0.69).

### 3.2. Predictors for Injury

**Figure 1:**
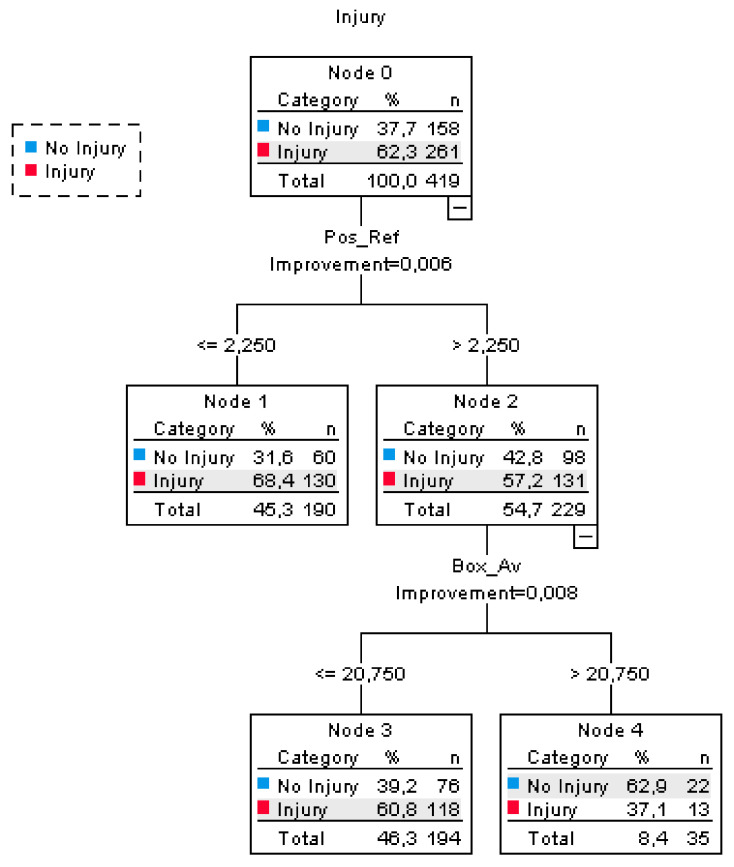
The results of the Classification and Regression Trees analysis showing the four terminal nodes. Predictors included in the CRT analysis were: perceived stress, square hop test, One-leg Long Box Jump Test (OLLBJ), active coping, instrumental support, planning, acceptance, emotional support, positive reframing, humor, religion, number of training hours/week, number of match hours/week, age.

The results from the CRT decision-tree analysis presented a solution with two parent nodes and four terminal nodes (see Figure 1). The coping strategy “positive reframing” was found to be the main predictor of traumatic injuries among adolescent female soccer players. More specifically, players with lower levels of positive reframing (≤2.25) were exposed to an increased risk of sustaining at least one traumatic injury in comparison to the players with higher levels of positive reframing (Risk difference = 11.2%, 95% CI = [2.01, 20.42]). Among the players who reported higher levels of positive reframing the performance on the Square hoptest was, in turn, a predictor of injuries. A higher number of square jumps (>20.75/15 s.), indicating high performance in this functional test, were for these persons associated with an even more-reduced risk of traumatic injuries (Risk difference = 23.7%, 95% CI = [6.26, 41.10]). Neither of the other proposed predictors reached the prespecified threshold relative reduction to error.

## 4. Discussion

Based on previous suggestions about the interdisciplinary combination of risk factors for sport injuries, this study aimed to investigate if demographic factors, coping strategies, perceived stress, and functional performance were predictors of traumatic injuries in young female football players. The results showed that 62% of the players reported at least one traumatic injury during the 52 weeks. The strongest predictor of traumatic injuries was the coping strategy “positive reframing”. The main finding was that the combination of high levels of positive reframing and high levels of physical performance capacity decreased the risk of injury. More specifically, the combination of certain psychological strategies and physiological skills to handle different types of stressors (e.g., psychological, physiological) are likely to predict who does not get injured. This is in line with the suggestions that it is a nonlinear combination of different risk factors that will determinate the injury risk an athlete is exposed to [23].

These results are also in line with results from several multifactorial models that include intrinsic factors such as, e.g., neuromuscular control [25] and psychological factors [16,24]. These models suggest that an athlete who is experiencing extrinsic risk factors, e.g., rules, referees, weather, or opponents, is predisposed for injury. For being susceptible to injuries, the interaction of any of the risk factors is suggested to increase the risk. The interactions of intrinsic and extrinsic factors accumulate the risk for injury [25]. The results of the present study indicate that neuromuscular control and adaptive coping strategies are buffers for traumatic injuries and prolong the accumulation of predictors. A football player who is able to land with control after a heading or jumping possesses an important skill that can protect from injuries in the lower extremities in such situations.

The results showed that positive reframing was the psychological factor that had the strongest relationship with injury. More specially, lower levels of positive reframing were associated with increased risk of traumatic injuries. Positive reframing is a beneficial emotional coping strategy where the person uses cognitive transformation to facilitate positive emotions and calming down when facing a stressful situation [40]. This type of coping strategy is related to, for example, lower levels of depression symptoms (e.g., Gurvich et al., 2020 [41]). Depressive symptoms can, in turn, increase the risk of injury via, for example, increased magnitude of physiological and attentional stress responses (e.g., Yang et al., 2014) [42]. Because female athletes report, in general, higher prevalence of depressive symptoms in comparison to male athletes [43] this coping strategy might be extra relevant for the study population.

The score on the functional performance test was the other factor that predicted injury. One potential explanation is that the most common injuries in female football are traumatic injuries in the lower extremities [4]. Hence, neuromuscular injury prevention programs do include exercises aiming to increase functional performance when landing or moving quickly in different directions, e.g., injuries without contact with others. Functional performance tests are thereby useful to evaluate an athlete’s dynamic alignment (e.g., body control, balance, and stability); modifiable skills that are important to avoid injuries [44]. A history of injuries is one of the most established risk factors for injuries [45]. The reason for this may vary, however a completed rehabilitation evaluated with functional performance tests showed decreased risk for reinjuries [46,47] and reaching preinjury level when return to sport [48].

### 4.1. Methodological Considerations

The strength of this study was the novelty of our research, comprising several domains which allow for investigating potential complex patterns of injury mechanisms. The included domains correspond to some of the previously suggested internal risk factors [16,24,25], representing a biopsychosocial approach [15]. The longitudinal study design with a large sample of adolescent female football players is another strength. The response rate during the 52 weeks was 74% of all reports, which implies that we were able to capture injuries, physical complaints, and related data to a large extent. This group of footballers is important to study, because injuries are common, and to remain injury-free may contribute to players being physically active for a longer period.

One potential limitation was that we did not collect information about external factors which may moderate the relationships between several risk factors and the risk of injury. Another limitation was that the potential predictors only were collected at baseline. Given that both physiological and psychosocial factors might change over time, the levels of the different factors might have changed before the potential injury occurred. Finally, we have used a biopsychosocial perspective but have not been able to give equal importance to all three parts of such perspective.

### 4.2. Implications

Participants with lower levels of positive reframing experienced an increased risk of sustaining one traumatic injury in comparison to the players with higher levels of positive reframing. Emotion-focused coping strategies such as, e.g., positive reframing, are suggested to increase hardiness and stress-related growth after sports injuries [49]. Coping strategies are modifiable, and coaches and supporting staff are therefore encouraged to increase adaptive coping skills, i.e., positive reframing, in young female football players through education and psychological skills training.

## 5. Conclusions

The combination of more frequent use of the coping strategy of positive reframing and high levels of physical performance capacity may prevent a traumatic injury in adolescent female footballers. Coaches are encouraged to adopt both physiological and psychological factors when preventing injuries in young female footballers.

## Figures and Tables

**Table 1:** The included players’ demographic information.

Variable	Females (*n* = 419)
Age year, mean (SD)	13.9 (1.1)
Years of playing football, mean (SD)	7.0 (2.2)
Training hours/week, mean (SD)	5.0 (1.8)
Match/week, mean (SD)	1.5 (0.6)
Injured players last 2 months prior to baseline, *n* (percent)	200 (48)
Injured players (≥1 traumatic injury during study, 52 weeks), *n*, (percent)	261 (62)

**Table 2:** Results of the descriptive analysis of the independent variables (stress, functional tests, and coping strategies).

Variable	M (SD)	Correlations										
		1	2	3	4	5	6	7	8	9	10	11
1. Stress	2.00 (1.08)	1	−10 *	−0.01	−0.17 *	−0.06	−0.11 *	−0.01	0.08	0.13	−0.20 *	0.02
2. OLLBJ	3.95 (1.04)		1	0.31 *	0.05	0.02	−0.01	−0.06	−0.02	0.07	0.07	0.09
3. Sq hop	17.10 (3.44)			1	0.05	0.05	−0.02	0.03	0.02	−0.02	0.02	0.05
4. AC	2.97 (0.73)				1	0.43 *	0.50 *	0.13 *	0.36 *	−0.08	0.34 *	−0.01
5. I Supp	2.78 (0.86)					1	0.30 *	0.19 *	0.71 *	0.02	0.35 *	0.05
6. Plan	2.64 (0.78)						1	0.25 *	0.24 *	0.03	0.37 *	0.09
7. Acc	2.68 (0.74)							1	0.22 *	0.18 *	0.28 *	0.06
8. Em Supp	2.90 (0.87)								1	0.07	0.35 *	−.02
9. Humor	1.91 (0.86)									1	0.06	0.02
10. Pos Ref	2.36 (0.81)										1	0.12 *
11. Religion	1.14 (0.41)											1

Values are mean (M), and standard deviation (SD), * *p* < 0.05. Stress = perceived stress, OLLBJ = One-leg Long Box Jump Test Sq hop = square hop test, Brief COPE: AC = active coping, I Supp = instrumental support, Plan = planning, Acc = acceptance, Em Supp = emotional support, Pos Ref = positive reframing, number of training hours/week, number of match hours/week, Age.

## Data Availability

The dataset generated and analyzed for this manuscript is part of a larger data collection which is not finalized. Data can be available upon request.

## References

[1] Larruskain J., Lekue J.A., Diaz N., Odriozola A., Gil S.M. (2018). A comparison of injuries in elite male and female football players: A five-season prospective study. Scand. J. Med. Sci. Sports.

[2] López-Valenciano A., Raya González J., Garcia Gómez A., Aparicio Sarmiento A., Sainz de Baranda P., De Ste Croix M., Ayala F. (2021). Injury profile in women’s football: A systematic review and meta-analysis. Sports Med..

[3] Bennett P., Fawcett L. (2006). Trauma injuries sustained by female footballers. Trauma.

[4] Clausen M.B., Kreutzfeldt Zebis M., Møller M., Krustrup P., Holmich P., Wedderkopp N., Andersen L.L., Bang Christensen K., Thorborg K. (2014). High injury incidence in adolescent female soccer. Am. J. Sports Med..

[5] Faude O., Junge A., Kindermann W., Dvorak J. (2005). Injuries in female soccer players—A prospective study in the German national league. Am. J. Sports Med..

[6] Hägglund M., Waldén M., Ekstrand J. (2009). Injuries among male and female elite football players. Scand. J. Med. Sci. Sports.

[7] Le Gall F., Carling C., Reilly T. (2008). Injuries in young elite female soccer players. Am. J. Sports Med..

[8] Hägglund M., Waldén M., Ekstrand J. (2006). Previous injury as a risk factor for injury in elite football: A prospective study over two consecutive seasons. Br. J. Sports Med..

[9] Alahmad T.A., Kearney P., Cahalan R. (2020). Injury in elite women’s soccer: A systematic review. Phys. Sportsmed.

[10] Hagglund M., Walden M. (2016). Risk factors for acute knee injury in female youth football. Knee Surg. Sports Traumatol. Arthrosc..

[11] Ostenberg A., Roos H. (2000). Injury risk factors in female European football. A prospective study of 123 players during one season. Scand. J. Med. Sci. Sports.

[12] Frisch A., Urhausen A., Seil R., Croisier J.-L., Windal T., Theisen D. (2011). Association between preseason functional tests and injuries in youth football: A prospective follow-up. Scand. J. Med. Sci. Sports.

[13] Smith J., DePhillipo N., Kimura I., Kocher M., Hetzler R. (2017). Prospective functional performance testing and relationship to lower extremity injury incidence in adolescent sports participants. Int. J. Sports Phys. Ther..

[14] Christopher R., Brandt C., Benjamin-Damon N. (2021). Systematic review of screening tools for common soccer injuries and their risk factors. S. Afr. J. Physiother..

[15] Appaneal R., Perna F., Eklund R., Tenenbaum G. (2014). Biopsychosocial Model of Injury. Encyclopedia of Sport and Exercise Psychology.

[16] Williams J.M., Andersen M.B. (1998). Psychosocial antecedents of sport injury: Review and critique of the stress and injury model. J. Appl. Sport Psychol..

[17] Ivarsson A., Johnson U., Andersen M.B., Tranaeus U., Stenling A., Lindwall M. (2017). Psychosocial factors and sport injuries: Meta-analyses for prediction and prevention. Sports Med..

[18] Ivarsson A., Johnson U., Lindwall M., Gustafsson H., Altemyr M. (2014). Psychosocial stress as a predictor of injury in elite junior soccer: A latent growth curve analysis. J. Sci. Med. Sport.

[19] Johnson U., Ivarsson A. (2011). Psychological predictors of sport injuries among junior soccer players. Scand J. Med. Sci Sports.

[20] Pensgaard A.M., Ivarsson A., Nilstad A., Solstad B.E., Steffen K. (2018). Psychosocial stress factors, including the relationship with the coach, and their influence on acute and overuse injury risk in elite female football players. BMJ Open Sport Exerc Med..

[21] Steffen K., Pensgaard A.M., Bahr R. (2009). Self-reported psychological characteristics as risk factors for injuries in female youth football. Scand. J. Med. Sci. Sports.

[22] Rice S., Purcell R., Silva S., Mawren D., McGorry P., Parker A. (2016). The mental health of elite athletes: A narrative systematic review. Sports Med..

[23] Bittencourt N., Meeuwisse W., Mendonça L., Nettel-Aguirre A., Ocarino J., Fonseca S. (2016). Complex systems approach for sports injuries: Moving from risk factor identification to injury pattern recognition—Narrative review and new concept. Br. J. Sports Med..

[24] Bahr R., Krosshaug T. (2005). Understanding injury mechanisms: A key component of preventing injuries in sport. Br. J. Sports Med..

[25] Meeuwisse W.H., Tyreman H., Hagel B., Emery C. (2007). A dynamic model of etiology in sport injury: The recursive nature of risk and causation. Clin. J. Sport Med..

[26] Alentorn-Geli E., Myer G.D., Silvers H.J., Samitier G., Romero D., Lázaro-Haro C., Cugat R. (2009). Prevention of non-contact anterior cruciate ligament injuries in soccer players. Part 2: A review of prevention programs aimed to modify risk factors and to reduce injury rates. Knee Surg. Sports Traumatol. Arthrosc..

[27] McBain K., Shrier I., Shultz R., Meeuwisse W.H., Klugl M., Garza D., Matheson G.O. (2012). Prevention of sports injury I: A systematic review of applied biomechanics and physiology outcomes research. Br. J. Sports Med..

[28] Crossley K.M., Patterson B.E., Culvenor A.G., Bruder A.M., Mosler A.B., Mentiplay B.F. (2020). Making football safer for women: A systematic review and meta-analysis of injury prevention programmes in 11,773 female football (soccer) players. Br. J. Sports Med..

[29] Ivarsson A., Stenling A., Balakrishnan N., Colton T., Everitt B., Piegorsch W., Ruggeri F., Teugels J. (2014). Prediction of Injury Risk in Sports. StatsRef: Statistics Reference Online.

[30] Von Elm E., Altman D.G., Egger M., Pocock S.J., Gotzsche P.C., Vandenbroucke J.P. (2014). The Strengthening the Reporting of Observational Studies in Epidemiology (STROBE) Statement: Guidelines for reporting observational studies. Int. J. Surg..

[31] Fuller C.W., Ekstrand J., Junge A., Andersen T.E., Bahr R., Dvorak J., Hagglund M., McCrory P., Meeuwisse W.H. (2006). Consensus statement on injury definitions and data collection procedures in studies of football (soccer) injuries. Br. J. Sports Med..

[32] Salminen S., Kouvonen A., Koskinen A., Joensuu M., Väänänen A. (2014). Is a single item stress measure independently associated with subsequent severe injury: A prospective cohort study of 16,385 forest industry employees. BMC Public Health.

[33] Carver C.S. (1997). You want to measure coping but your protocol’s too long: Consider the brief COPE. Int. J. Behav. Med..

[34] Caffrey E., Docherty C.L., Schrader J., Klossner J. (2009). The ability of 4 single-limb hopping tests to detect functional performance deficits in individuals with functional ankle instability. J. Orthop. Sports Phys. Ther..

[35] Sharma N., Sharma A., Sandhu J.S. (2011). Functional performance testing in athletes with functional ankle instability. Asian J. Sports Med..

[36] Clarsen B., Myklebust G., Bahr R. (2013). Development and validation of a new method for the registration of overuse injuries in sports injury epidemiology: The Oslo Sports Trauma Research Centre (OSTRC) overuse injury questionnaire. Br. J. Sports Med..

[37] Ekman E., Frohm A., Ek P., Hagberg J., Wiren C., Heijne A. (2015). Swedish translation and validation of a web-based questionnaire for registration of overuse problems. Scand. J. Med. Sci. Sports.

[38] Machuca C., Vettore M.V., Krasuska M., Baker S.R., Robinson P.G. (2017). Using classification and regression tree modelling to investigate response shift patterns in dentine hypersensitivity. BMC Med. Res. Methodol..

[39] Venkatasubramaniam A., Wolfson J., Mitchell N., Barnes T., JaKa M., French S. (2017). Decision trees in epidemiological research. Emerg. Themes Epidemiol..

[40] Stanisławski K. (2019). The coping circumplex model: An integrative model of the structure of coping with stress. Front. Psychol..

[41] Gurvich C., Thomas N., Thomas E.H., Hudaib A.R., Sood L., Fabiatos K., Sutton K., Isaacs A., Arunogiri S., Sharp G. (2021). Coping styles and mental health in response to societal changes during the COVID-19 pandemic. Int. J. Soc. Psychiatry.

[42] Yang J., Schaefer J.T., Zhang N., Covassin T., Ding K., Heiden E. (2014). Social support from the athletic trainer and symptoms of depression and anxiety at return to play. J. Athl. Train..

[43] Golding L., Gillingham R.G., Perera N.K.P. (2020). The prevalence of depressive symptoms in high-performance athletes: A systematic review. Phys. Sportsmed..

[44] Dinc E., Kilinc B.E., Bulat M., Erten Y.T., Bayraktar B. (2017). Effects of special exercise programs on functional movement screen scores and injury prevention in preprofessional young football players. J. Exerc. Rehabil..

[45] Nilstad A., Andersen T.E., Bahr R., Holme I., Steffen K. (2014). Risk factors for lower extremity injuries in elite female soccer players. Am. J. Sports Med..

[46] Niederer D., Giesche F., Janko M., Niemeyer P., Wilke J., Engeroff T., Stein T., Frank J., Banzer W., Vogt L. (2020). Unanticipated jump-landing quality in patients with anterior cruciate ligament reconstruction: How long after the surgery and return to sport does the re-injury risk factor persist?. Clin. Biomech..

[47] Bien D.P., Dubuque T.J. (2015). Considerations for late stage ACL rehabilitation and return to sport to limit re-injury risk and maximize athletic performance. Int. J. Sports Phys. Ther..

[48] Kitaguchi T., Tanaka Y., Takeshita S., Tsujimoto N., Kita K., Amano H., Kinugasa K., Tachibana Y., Natsuume T., Horibe S. (2020). Importance of functional performance and psychological readiness for return to preinjury level of sports 1 year after ACL reconstruction in competitive athletes. Knee Surg. Sports Traumatol. Arthrosc..

[49] Salim J., Wadey R., Diss C. (2016). Examining hardiness, coping and stress-related growth following sport injury. J. Appl. Sport Psychol..

